# Phytochemicals in Cancer Prevention and Therapy: Truth or Dare?

**DOI:** 10.3390/toxins2040517

**Published:** 2010-03-31

**Authors:** Maria Russo, Carmela Spagnuolo, Idolo Tedesco, Gian Luigi Russo

**Affiliations:** Institute of Food Sciences, National Research Council, 83100, Avellino, Italy; Email: mrusso@isa.cnr.it (M.R.); carmela.spagnuolo@gmail.com (C.S.); idolo@isa.cnr.it (I.T.)

**Keywords:** isothiocyanate, curcumin, genistein, epigallocatechin gallate, lycopene, resveratrol, glucosinolates, clinical trials, apoptosis, phytochemicals

## Abstract

A voluminous literature suggests that an increase in consumption of fruit and vegetables is a relatively easy and practical strategy to reduce significantly the incidence of cancer. The beneficial effect is mostly associated with the presence of phytochemicals in the diet. This review focuses on a group of them, namely isothiocyanate, curcumin, genistein, epigallocatechin gallate, lycopene and resveratrol, largely studied as chemopreventive agents and with potential clinical applications. Cellular and animal studies suggest that these molecules induce apoptosis and arrest cell growth by pleiotropic mechanisms. The anticancer efficacy of these compounds may result from their use in monotherapy or in association with chemotherapeutic drugs. This latter approach may represent a new pharmacological strategy against several types of cancers. However, despite the promising results from experimental studies, only a limited number of clinical trials are ongoing to assess the therapeutic efficacy of these molecules. Nevertheless, the preliminary results are promising and raise solid foundations for future investigations.

## 1. Introduction

To date, several hundred scientific studies focused on the activity of non-nutritional compounds present in the diet, preventing the occurrence of degenerative diseases, such as cancer. This heterogeneous class of molecules, generally known as phytochemicals includes vitamins (carotenoids) and food polyphenols, such as flavonoids, phytoalexins, phenolic acids indoles and sulfur rich compounds [1,2,3]. More than 10,000 phytochemicals have been described, and among them more than 6,000 compounds are included in the class of flavonoids [4]. They are widely present in plant derived foods and beverages (fruits, vegetables and beverage such as tea, wine beer and chocolate), and in many dietary supplements or herbal remedies. Due to the variety of their physiological roles in plant tissues in regulating enzymes involved in cell metabolism and in mechanisms of defence against foreign agents (radiations, viruses, parasites), phytochemicals have been associated to pleiotropic effects in animal cells. Phytochemicals attracted scientists’ interests since the demonstration that their biological targets in mammalian cells were the same involved in inflammatory processes and oncogenic transformation, such alterations of cell cycle control, apoptosis evasion, angiogenesis and metastases. In addition, a large number of epidemiological studies suggest that a daily intake of phytochemicals can reduce the incidence of several types of cancers [2,3,5,6]. However, we and others already underlined the discrepancy between phytochemical concentrations applied in *in vitro* studies (usually tens of micromolars) and those found *in vivo* (human and animal sera), after vegetable and fruit ingestion (usually below 1 μM) [1]. The very low concentrations of free aglycones found *in vivo* are due to the scarce bioavailability and biotransformation of these molecules discussed in excellent reviews [7,8]. In fact, many phytochemicals, including polyphenols, are rapidly degraded and metabolized in the human body. Moreover, genetic variation in pathways affecting absorption, metabolism, and distribution of these natural substances, could influence exposure at the tissue level, thus modifying disease risk in individuals [9,10]. Nevertheless, this wide group of natural molecules represents a promising class as anticancer drugs, since their multiple targets in cancer cells, with limited toxic effect on normal cells. Phytochemicals can prove their therapeutic efficacy in mono-treatments or in association with classical chemotherapeutic drugs. In the latter case, a double positive effect can be expected: i. phytochemicals can synergize with cytotoxic drugs, increasing their efficacy and lowering the toxic side effects on normal cells; ii. combined treatment can delay resistance onset.

Despite this encouraging preamble and the abundant literature describing the molecular mechanisms triggered by phytochemicals to inhibit cell growth and induce apoptosis in cancer cells, only few of them entered clinical trials. Here, we will focus our attention on a selection of representative molecules, namely isothiocyanate, curcumin, genistein, epigallocatechin gallate, lycopene and resveratrol, largely present in the literature. For each of them, we will summarize their putative mechanism(s) of action from *in vitro* and animal studies, and the current status of their clinical application in view of their realistic adoption as single chemotherapeutic agents or as chemosensitizers, in association with canonical and novel anti-cancer drugs.

## 2. Isothiocyanates

Fruits and vegetables are important sources of secondary compounds that may supply nourishing health benefits beyond basic nutrition; examples of the latter are the glucosinolate products, such as sulforaphane (Table 1). Glucosinolates occur as secondary metabolites of many plants of the *Brassicaceae* family. Plants use substances derived from glucosinolates as natural pesticides and as defense against herbivores; these compounds are also responsible for the bitter or sharp taste of many common foods such as cabbage, mustard, horseradish, and Brussels sprouts. Glucosinolates are water-soluble anions and belong to the class of glucosides. All glucosinolates contain a central carbon atom which is bond, *via* a sulfur atom, to the glycone group, and, via a nitrogen atom, to a sulfonated oxime group. In addition, the central carbon is bond to a side group which confers specific to the molecule, since each glucosinolate has a different side groups (Table 1) [11]. More than 120 glucosinolates are known to naturally occur in plants. They are synthesized from amino acids, such as methionine, phenylalanine, tyrosine or tryptophan. Plant enzyme myrosinase (β-thioglucoside glucohydrolase), in the presence of water, hydrolyze the glucose group from glucosinolate leading to the quick formation of thiocyanates, isothiocyanates (ITCs) or nitriles. These are the active compounds employed by the plants against parasites. Even when plant myrosinase is completely inactivated by heat, the myrosinase activity of human intestinal bacteria allows the formation and absorption of ITCs [12]. However, the absorption and excretion of ITCs is substantially lower in cooked, compared to raw cruciferous vegetables [13].

ITCs are a heterogeneous family of molecules with the –N=C=S group as their common structural feature (Table 1). Unlike the glucosinolates, which are largely chemically stable and biologically inert, ITCs are electrophilic and biologically active. Vegetables contain different glucosinolate precursors, such as sinigrin (allyl isothiocyanate; AITC) in broccoli and Brussels sprouts; glucotropaeolin (benzyl isothiocyanate; BITC) in cabbage, gluconasturtiin (phenethyl isothiocyanate, PEITC) in watercress; and glucoraphanin (sulforaphane) (Table 1) in broccoli, Brussels sprouts, cabbage.

A limited number of reports have dealt with their bioavailability [14]. To establish the blood concentration of sulforaphane in male rats after oral administration, the pharmacokinetics of sulforaphane was determined after a dose of 50 μmol. The plasma drug concentrations occurred at 1 h and increased to a peak of around 20 μM at 4 h after dosing. This concentration of sulforaphane offers clear relevance for numerous *in vitro* cell culture studies, where sulforaphane was typically employed at 1–30 μM on a variety of *in vitro* signal transduction studies as well as phase II gene induction studies [15]. In a human study, ITC levels were determined in healthy volunteers after intake of vegetables. Sulforaphane levels were attained 1 h after consuming a broccoli soup with the molecule remaining still detectable in human plasma 24 h after [16]. Even if it is reasonable that repeated intake of vegetables may lead to higher plasma levels of ITCs, increasing its biological activity, Hanlon demonstrated that reiterated intake of broccoli does not determine sulforaphane accumulation in blood [17]. Dietary supplements containing extracts of broccoli sprouts, broccoli, and other cruciferous vegetables are available without a prescription. Some products are standardized to contain a minimum amount of glucosinolates and/or sulforaphane. However, the bioavailability of ITCs derived from these supplements is currently unknown.

**Table 1:** Chemical structure of selected phytochemicals ^1^.

Toxin name	Chemical structure
Glucosinolate	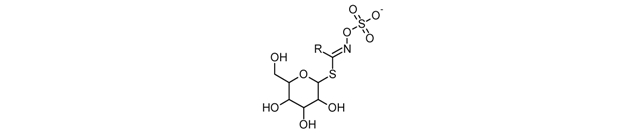
Sulforaphane	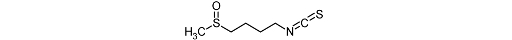
Curcumin	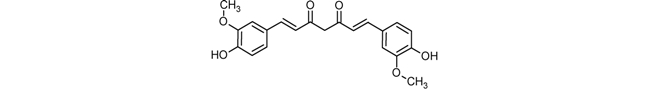
Genistein	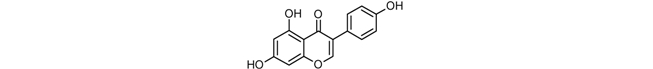
Phenoxodiol (Synthetic genistein analog)	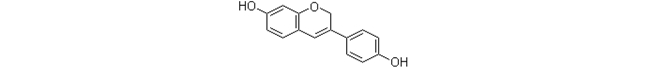
Epigallocatechin gallate (ECGC)	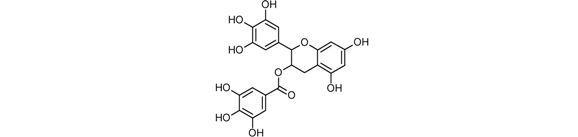
Lycopene	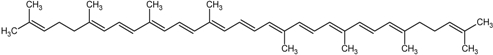
Resveratrol	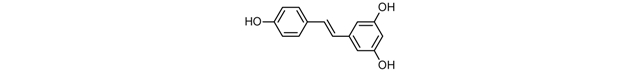

^1 ^Structure were prepared using ChemSketch Freeware 12.0 from Advanced Chemistry Development (www.acdlabs.com).

Several animal studies of chemical induced carcinogenesis demonstrated that ITCs are able to inhibit cancer development (pre-initiation) [18]. However, very high intakes of PEITC or BITC (25–250 times higher than the average human intake of dietary ITCs) have been found to promote bladder cancer in rats when given after a chemical carcinogen (post-initiation) [19]. The relevance of these findings to human urinary bladder cancer is not clear, since at least one prospective cohort study found cruciferous vegetable consumption to be inversely associated with the risk of bladder cancer in men [20]. Many relevant biological effects of ITCs have been elucidated with respect to its anticancer properties. ITCs modulate events linked to clonal expansion in leukemia transformed cells, such as cell-cycle progression, apoptosis, and induction of differentiation. As an example, sulforaphane, at 4 μM concentration, caused 85% inhibition in U937 cell growth [21]. Two of the most abundant glucosinolates, sinigrin and gluconasturtin, which are hydrolyzed to AITC and PEITC, respectively, inhibited, in a dose-dependent manner, cell growth in HL-60 cell line (p53-null) with an IC_50_ of 2.56 and 1.49 μM, respectively. At 5 μM concentration, PEITC induced caspase-8 activation in HL-60, during the initial 3 h of treatment [22]. BITC inhibited the benzo[*a*]pyrene-induced lung tumorigenesis in A/J mice [23], and reduced dramatically WEHI-3 leukemia cell viability with a sub-micromolar IC_50_, by causing G0/G1 arrest followed by apoptosis and caspase-3 activation [24]. Sulforaphane differentiated HL-60 into both granulocytes and macrophages, possibly by the involvement of PI_3_K/PKC [25].

ITCs are effective cancer chemopreventive agents [26]. In this contest, they generally act triggering detoxifying enzymes, thus blocking the initiation of carcinogenesis [27]. More recently, it has been demonstrated that oral feeding of synthetic isothiocyanate PHI (phenylhexyl isothiocyanate), at a dose of 12 μmol/day, significantly reduced tumor formation in immunodeficient mice, with xenografts of human leukemia HL-60 cell, with no evidence of particular toxicity to normal tissue cells [28]. This work suggests that PHI may also act at the post-initiation level. The smaller weight of PHI-treated tumor was due to apoptosis. PHI inhibited the *in vivo* cell cycle progression through the down regulation of cyclin expression [28]. Many chemotherapeutic drugs, such as arsenic trioxide (ATO), possess a limited activity as a single agents in several hematological malignancies [29]. In a recent paper, incubation of leukemic cells in the presence of sulforaphane (3 μM) combined with ATO (0.3 μM) strongly enhanced ATO-mediated apoptosis [30]. Combination of ATO/sulforaphane therapy resulted in a significant intracellular ROS (Reactive Oxygen Species) increase.

Despite substantial progress in the understanding of the molecular basis of anticancer activities of ITCs, very limited clinical studies on cancer patients have been reported. A phase I clinical trial is currently underway (Table 2). The goal of this study, approved but not yet active, is to determine the highest tolerable dose of PEITC to be administered to patients affected by lymphoproliferative disorder and treated with fludarabine.

Finally, it must be mentioned that for some ITC compounds tumor promoting and carcinogenic activities have been described. As an example, oral administration PEITC and BITC induced continuous urinary epithelial cell proliferation leading to bladder carcinogenesis in rats. The induction of bladder lesions may arise from the hydrophobic activity of ITCs, depending on the alkyl carbon chain length [31]. A possible cytotoxic and genotoxic mechanism of ITCs in the urinary bladder involves the formation of intracellular ROS from N=C=S group and subsequent oxidative DNA damage [32].

**Table 2:** Ongoing clinical trials testing dietary phytochemicals ^1^.

Trial Lead Organizations	Therapy		Phase		Type of cancer
	Polyphenol	Other treatments			
University of Texas - M.D. Anderson Cancer Center	Phenethyl isothiocyanate	Fludarabine	I	Active	Lymphoproliferative disorders
Mayo Clinic Cancer Center	17-AAG ^2^	Gemcitabine	II	Active	Recurrent Advanced Ovarian Epithelial or Primary Peritoneal Cavity Cancer
Mayo Clinic Cancer Center	17-AAG ^2^	Bortezomib	I	Active	Advanced solid tumors and lymphomas
Oregon Health and Science University - Knight Cancer Institute	Broccoli sprout extract		II	Active	Ductal carcinoma *in situ* and/or atypical ductal hyperplasia
University of Texas - M. D. Anderson Cancer Center	Curcumin		II	Active	Advanced pancreatic cancer
Rambam Medical Center	Curcumin	Gemcitabine	II	Active	Pancreatic cancer
Tel-Aviv Sourasky Medical Center	Curcumin	Gemcitabine and celecoxib	III	Active	Advance or inoperable pancreatic cancer
University of Texas - M. D. Anderson Cancer Center	Curcumin	Radiotherapy and capecitabine	II	Active	Rectal cancer
Tata Memorial Hospital	Curcumin	Ashwagandha extract^3^	I/II	Active	Advanced osteosarcoma
Cleveland Clinic Taussig Cancer Center	Curcumin	*Boswellia serrata*^4^	II	Active	Newly diagnosed or recurrent high-grade gliomas
University of Texas - M. D. Anderson Cancer Center	Curcumin	Bioperine		Completed	Multiple Myeloma
Johns Hopkins University	Curcumin		II	Terminated	FAP^5^
Memorial Sloan-Kettering Cancer Center	Green Tea Extract		I	Completed	Advanced solid tumors
Louisiana State University Health Sciences - Feist-Weiller Cancer Center	Green Tea Extract		II	Active	Breast cancer progression
UCLA - Jonsson Comprehensive Cancer Center	Green Tea - Black tea		II	Active	Prostate adenocarcinoma
University of Texas - M. D. Anderson Cancer Center	Polyphenon E ^6^		I	Closed	Breast cancer
University of Wisconsin - Paul P. Carbone Comprehensive Cancer Center	Polyphenon E ^6^		II	Active	Bladder Cancer
Barbara Ann Karmanos Cancer Institute	Polyphenon E ^6^		II	Active	MGUS^7^ and Multiple Myeloma
Mayo Clinic Cancer Center	Polyphenon E ^6^		II	Active	Chronic Lymphocytic Leukemia or Small Lymphocytic Lymphoma
Louisiana State University Medical Center - Stanley S. Scott Cancer Center	Polyphenon E ^6^	Erlotinib^8^	I/II	Active	NSLC ^9^
Aker Universitetssykehus HF	Syntetic Genistein (Bonistein)		II	Closed	Prostate cancer
Northwestern University - Robert H. Lurie Comprehensive Cancer Center	Genistein		II	Closed	Prostate cancer
Barbara Ann Karmanos Cancer Institute	Genistein	Gemcitabine	II	Completed	Breast cancer
University of North Carolina - Chapel Hill - Lineberger Comprehensive Cancer Center	Genistein		I	Closed	Prostate Cancer
Robert H. Lurie Comprehensive Cancer Center at Northwestern University	Genistein	Interleukin-2	II	Closed	Metastatic Melanoma or kidney cancer
University of Wisconsin Paul P. Carbone Comprehensive Cancer Center	Genistein		II	Closed	Bladder cancer
Barbara Ann Karmanos Cancer Institute	Genistein	Gemcitabine, and Erlotinib	II	Closed	Pancreatic Cancer
University of Minnesota - Masonic Cancer Center	Genistein		I/II	Active	Osseous Metastases
Parker Hughes Cancer Center	Genistein immunoconjugate		I	Closed	Recurrent B-cell Acute Lymphoblastic Leukemia or NHL ^10^
Barbara Ann Karmanos Cancer Institute	Soy isoflavones		II	Closed	Adenocarcinoma of the Prostate
H. Lee Moffitt Cancer Center CCOP Research Bas	Soy isoflavones		NS	Closed	Breast cancer
Barbara Ann Karmanos Cancer Institute	Soy isoflavones	Radiotherapy	II	Closed	Prostate cancer
Wake Forest University Comprehensive Cancer Center	Soy isoflavones	Cholecalciferol	II	Active	Prostate cancer
University of South Florida - H. Lee Moffitt Cancer Center and Research Institute	Purified isoflavones		II	Active	Prostate Cancer
Novogen, Incorporated	Phenoxodiol ^11^		I	Completed	Refractory solid tumors
Yale Cancer Center	Phenoxodiol ^11^	Docetaxel	I/II	Active	Ovarian epithelial, fallopian tube or primary peritoneal cavity cancer
Marshall Edwards, Inc.	Phenoxodiol ^11^		II	Active	Prostate cancer
University of Texas - M. D. Anderson Cancer Center	Lycopene		II	Closed	Prostate cancer
H. Lee Moffitt Cancer Center and Research Institute	Lycopene			Completed	Prostate cancer
University of Illinois Cancer Center	Lycopene		NS	Closed	Prostate cancer or benign prostatic hyperplasia
North Central Cancer Treatment Group	Lycopene		II	Completed	Metastatic prostate cancer
USC/Norris Comprehensive Cancer Center and Hospital	Lycopene	Supplements^12 ^	II	Active	Recurrent prostate cancer
Toronto Western Hospital	Lycopene	Antioxidants^13^	II	Active	Early stage prostate cancer
Toronto Western Hospital	Lycopene	Antioxidants^13^	II	Active	Prostate cancer
Ohio State University Medical Center - Arthur G. James Cancer Hospital and Solove Research Institute	Tomato-Soy Juice		I/II	Active	Prostate cancer
Chao Family Comprehensive Cancer Center at University of California Irvine Medical Center	Resveratrol		I/II	Closed	Colon Cancer
University of Michigan - Comprehensive Cancer Center	Resveratrol		I	Active	Resectable colorectal cancer
GlaxoSmithkline	SRT501^14^		I	Active	Colorectal cancer and hepatic metastases
GlaxoSmithkline	SRT501^14^	Bortezomib	II	Active	Multiple Myeloma

^1 ^Deducted primarily from http://www.cancer.gov/clinicaltrials; ^2 ^17-N-allylamino-17-demethoxygeldanamycin; ^3 ^Ingredients from traditional Indian medicine with potent anti-cancer compounds in laboratory as well as animal studies; ^4 ^Extracts of *Boswellia serrata* are used in traditional herbal medicine in India and in African countries. Animal experiments show anti-inflammatory activity of the extract; ^5 ^Familial Adenomatous Polyposis; ^6 ^A decaffeinated green tea catechin mixture containing about 50% EGCG and 30% other catechins; ^7 ^Monoclonal Gammopathy of Undetermined Significance; ^8 ^EFGR tyrosine kinase inhibitor; ^9 ^Non-Small Cell Lung Cancer; ^10 ^Non-Hodgkin's Lymphoma; ^11 ^Synthetic genistein; ^12 ^contains vitamin D3, vitamin E, selenium, green tea extract, saw palmetto, lycopene, and soy derivatives; ^13 ^vitamin E, selenium and lycopene; ^14 ^SRT501 is a proprietary chemical developed by Sirtris based on the naturally occurring resveratrol; NS, not specified.

## 3. Curcumin

Curcumin is a polyphenol (bis-α,β-unsaturated β−diketone, commonly called diferuloylmethane) derived from the rhizome of *Curcuma longa* (Table 1), also called turmeric, which belongs to the Zingiberaceae family. This perennial herb is cultivated in Asiatic countries, mainly in India and China. The yellow-pigmented fraction of turmeric contains curcuminoids: curcumin (curcumin I, that is the main component), demethoxycurcumin (curcumin II), bisdemethoxycurcumin (curcumin III), and cyclocurcumin. Curcuminoids are chemically related and represent 3%–5% of turmeric [33]. Traditionally, turmeric and natural curcuminoids have been used in folk medicine for its therapeutic effects, like antioxidant, anti-inflammatory, anticancer, antimicrobial, hepatoprotective, thrombosuppressive, hypoglycemic, antiarthritic. These therapeutic effects have been investigated and partially confirmed [34,35]. The anticancer properties of curcumin originate from several papers showing that the molecule can induce apoptosis in different cancer cell lines and inhibit the formation of tumours in animal models of carcinogenesis. The anti-carcinogenic activities of curcumin have been attributed to its effects on several targets including transcription factors, growth factors and their receptors, cellular signaling molecules, genes regulating cell proliferation and apoptosis, adhesion molecules and regulators of angiogenesis [36]. These pleiotropic activities derive from the complex chemistry of the molecule (unsaturation in the side chain methoxy groups in the benzene ring and central β-diketone moiety) which influences multiple signaling pathways by direct interaction or through modulation of gene expression [37].

The anti-carcinogenic effects of curcumin on pancreatic cancer have been initially demonstrated in a variety of pancreatic cell lines, where the molecule suppresses proliferation and induces apoptosis through down-regulation of constitutively active NF-κB and IκB kinase [38]. In pancreatic cell line, curcumin also inhibits cell growth by down-regulation of COX-2, EGFR, ERK1/2 [39]. The *in vitro* anticancer properties have been also confirmed in *in vivo* studies. In murine xenograft models, pancreatic cancer cells were subcutaneously injected into nude mice and intravenous administration of liposomal curcumin caused reduction of tumor size and decrease of CD31, VEGF and IL-8 expression [38]. In addition, a combination of curcumin and gemcitabine inhibited pancreatic cancer growth in nude mice better than gemcitabine alone, suggesting chemosensitizing effect of the molecule [40]. A large number of clinical trials have been performed to investigate the pharmacokinetics, safety and efficacy of curcumin, to show its therapeutic potential against different cancers including leukemia and lymphoma, gastrointestinal cancer, genitourinary cancer, breast cancer, pancreatic cancer, lung cancer, melanoma, neurological cancer and others [36,41]. Clinical trials on patients affected by advanced pancreatic cancer, familiar adenomatous polyposis (FAP), and multiple myeloma (MM) are obtaining promising results (Table 2). Curcumin efficacy in the treatment of human pancreatic cancer has been reported in a phase II clinical trial in patients affected by advanced pancreatic cancer [42]. Twenty-five patients were enrolled and twenty-one were evaluable for response. A dose of 8 g/day of curcumin was administered orally until disease progression, with restaging every 2 months. In PBMC (peripheral blood mononuclear cells) of patients who received curcumin has been noted down-regulated expression of NF-κB, COX-2 and phosphorylated STAT3. In two patients, antitumor effects of curcumin was observed; one had ongoing stable disease for more than 18 months and one had 73% tumor reduction, but he was short-lived. In this latter patient, a rapid and dramatic increase in serum cytokine levels (release of cytokines from the tumor has associated with shrinkage) was detected. This study showed that oral curcumin is well tolerated without toxicity at doses of 8 g/day for up to 18 months. Low levels of curcumin was measured in the plasma (steady-state level at day 3 was 22–41 ng/mL), but, although the molecule was poorly absorbed, a limited number of patients (9.5%) beneficed of the positive effects of curcumin [42]. It is well-known that curcumin *in vitro* show antiproliferative effects when used to micrograms amounts [38]. Therefore, the limited bioavailability of curcumin probably attenuated the response rate.

A significant number of studies describe the antiproliferative mechanism of curcumin against MM. In four different MM cell lines (U266, RPMI 8226, MM.1, and MM.1R), curcumin blocked the constitutively active IKK and inhibited NF-κB activation, leading to the suppression of proliferation and induction of apoptosis [43]. Moreover, the molecular consequence of NF-κB suppression by curcumin was the reduced expression of several gene products regulated by NF-κB, such as IκB, Bcl-2, Bcl-X_L_, IL-6, and cyclin D1 [43]. In other MM cells, curcumin inhibited IL-6 induced STAT3 phosphorylation and consequent STAT3 nuclear translocation, playing an important role in the suppression of MM proliferation [44]. Curcumin can overcome chemoresistance and enhance the activity of thalidomide and bortezomib, used to treat patients with MM, both *in vitro* and in a xenograft model in nude mice [45]. These results on experimental models have been confirmed in a promising phase I/II study where curcumin was administered to MM patients at growing concentration of 2, 4, 8, 12 g/day, orally. All doses were well tolerated without adverse events. Curcumin inhibited constitutive activation of NF-κB, COX-2 and STAT3 in the PBMC isolated from MM patients. 12 out of 29 patients continued the treatment for 12 weeks and five of them completed one full year of therapy with stable disease [46].

Several studies on a variety of colorectal cell lines indicate that curcumin might be employed as therapeutic agent against several molecular targets. As largely reviewed by others, in these cellular model, curcumin inhibits NF-κB activation, neurotensin-mediated activator protein-1, Ca^2+^ mobilization, PGE-2 (prostaglandin E2) and epidermal growth factor receptor (EGF-R) and down-regulates COX-1/2, MMP-2/9, IL-8 gene induction and colon cancer cell migration [47,48]. The potential use of curcumin as chemotherapic agent against colorectal neoplasia is also supported by animal studies and human clinical trials. In murine models induced with azoxymethane, curcumin inhibited the incidence of colonic adenocarcinomas and adenomas [49]. In Min mice, a murine model of FAP, curcumin significantly decreased tumor formation of 64% [50]. Oral administration of curcumin in cancer patients resulted in an increased concentration in colon tissue to approximately 10 μmol/kg, suggesting that oral curcumin can induce apoptosis in the gastrointestinal tract efficiently [51]. In a positive, but small clinical trial, the ability of curcumin to induce adenoma regression in five patients with FAP previously colectomized was evaluated [52]. The authors used an oral combination treatment including 480 of curcumin and 20 mg of quercetin three times a day for a mean duration of six months. All five patients had a significantly decreased number and size of ileal and rectal adenomas from baseline (60.4% and 50.9%, respectively), without producing any appreciable toxicity. The authors concluded that the putative anticancer mechanism(s) of curcumin in adenoma regression in FAP patients probably include up-regulation of carcinogen-detoxifying enzymes such as glutathione S-transferases, antioxidation, and suppression of the isoenzyme COX-2, and decrease in inducible PGE-2 [52].

There anticancer properties of curcumin are reinforced by the observation that, in many cases, the molecule kill tumor cells without damaging normal cells [53]. However, others suggest that curcumin may cause toxicity under specific conditions. A possible mechanism is that low concentrations of curcumin induce antioxidant effects, higher concentrations of this compound increase the cellular levels of ROS [54,55]. The main problem in the therapeutic use of the molecule remains its limited bioavailability. The low plasma and tissue levels of curcumin appear to be due to poor absorption, rapid metabolism, and rapid systemic elimination. To improve the bioavailability of curcumin, numerous approaches have been evaluated: (i). combination with an adjuvant like piperine, which interferes with glucuronidation; (ii). liposomal curcumin; (iii). curcumin nanoparticles; (iv). curcumin phospholipids complex; v. structural analogs of curcumin [56].

Table 2 reports clinical trials in active phase where curcumin is tested in monotherapy or combined with natural extracts in the treatment of pancreatic cancer, colorectal cancer, FAP, MM, glioma and osteosarcoma. Hopefully, results of these studies will be shortly available and will provide further evidence of the therapeutic efficacy of the molecule.

## 4. Genistein

Isoflavones, a sub-class of flavonoids, have a limited distribution in nature, and soyfoods are the only nutritionally relevant dietary source of these phytochemicals especially in Asian population where they provide 10% of total individual protein intake. There is a growing body of epidemiological studies suggesting that genistein (4',5,7-trihydroxyisoflavone) the predominant isoflavone in the plant family Leguminosae, which includes the soybean, may be helpful in preventing and treating several types of cancers, principally breast and prostate cancers [57,58,59]. Several mechanisms for the *in vitro* anti-cancer effects of genistein have been proposed [60]. Since the molecular structure of genistein (Table 1) and related compounds closely resembles those of estrogens, this group of molecules is also known as “phytoestrogen”. Genistein competes with 17-β-estradiol (E2) in Estrogen Receptor (ER) binding assays [61]. Its binding affinity is higher (87%) for ERβ than for ERα. ERs mediate the actions of hormone in different estrogen-dependent tissues, such as uterus, ovary and breast, where the levels and ratios of the two receptors are known to vary substantially. For example, ERα is the predominant form found in uterus and liver, whereas ERβ is mainly expressed in ovary. ERs also mediate the actions of estrogens and selective ER modulators in breast cancers, where 70% express ERβ form. The presence of ERα is associated with the proliferative effects of estrogens, whereas it is known that ERβ possesses growth-suppressive activities.

*In vitro* studies demonstrated that genistein inhibits growth of most types of hormone-dependent and -independent cancer cells and its effects on cellular signaling are dose depending [62]. In fact, it has been reported that preferential activation of ERβ by genistein is lost when genistein is increased from low (6 nM) to higher concentrations. At 300 nM, genistein activates both ERs; therefore, the final effect on gene expression and cell fate depends on ligand dose, and on the differential ability of ligand-ER complexes to recruit modulators at the ER binding sites of hormone-regulated genes [62]. Reasonably, the antiproliferative activity of genistein at concentrations higher than 10 μM is mediated by tyrosine kinase (PTK) inhibition. In fact, genistein is a potent *in vitro* inhibitor of PTKs activity, especially EGF-R [63]. This led to the suggestion that genistein might exert *in vivo* anticancer effects by inhibiting the activity of EGF-R PTK, or those of other PTKs, such as HER-2 [64]. In addition, similarly to other flavonoids (e.g., quercetin), genistein triggers regulative pathways under the control of MAPK, AKT and NF-κB [60]. Cell culture data indicate that genistein inhibits prostate specific antigen expression (PSA) in androgen-dependent and independent prostate cancer cell lines [65] supporting the role of genistein as a chemopreventive/therapeutic agent for prostate cancer irrespective of androgen responsiveness.

Both *in vitro* and epidemiological studies showing anticancer properties of genistein have been confirmed by *in vivo* data obtained on animal models. These studies utilize genistein in different formulas. Standard soy isoflavone formula contains genistein mainly in the form of genistein (genistein monoglycosides), as well as other isoflavones (daidzin and glycitin). The percentage of different isoflavones present in this soy formula correlates with the amount of the same compounds found in soybeans, *i.e.*, 50% genistein, 38% daidzin, and 12% glycitin. A 50 mg dose of soy isoflavones (a typical daily dose) provides 25 mg of genistein, 19 mg of daidzin and about 6 mg of glycitin. Pharmacologic research has developed also synthetic genistein analogs. The most representative are 1-bonistein and 2-phenoxodyol. 1-Bonistein is a pure synthetic genistein, developed by DSM Nutritional Products (Basel, Switzerland) consisting in 99.4% synthetic genistein aglycone. It has been tested in healthy volunteers in phase I clinical trial to assess safety and tolerability [66]. Tolerability of different doses (from 30 to 300 mg) was good and uptake was also very rapid, as revealed by plasma concentration-time course. Mean C_max_ values of 252.0, 605.0, 1,518.0, and 1,808.0 ng/mL were observed in the 4–6 h range. Bonistein is actually evaluated in an active clinical trial in patients who are undergoing surgery for prostate cancer (Table 2). 2-Phenoxodyol is a synthetic isoflavone with 1,2 diphenylpropane-ring structure (Table 1) characterized by a more pronounced antineoplastic activity with reduced toxicity in cancer-derived cell lines and animal models [67]. It was tested in phase I/II clinical trial by continuous intravenous infusion in patients with solid cancer. This study demonstrated that a seven-day continuous infusion of phenoxodiol given every two weeks showed no severe toxicity up to a dose of 27.0 mg/kg/day [68]. Moreover, a recent study employed phenoxodiol to sensitize chemoresistant ovarian cancer cells to platinum and taxane drugs, as well as gemcitabine and topotecan [67]. The importance of this study is underlined by the interest of FDA (Food and Drug Administration, USA) to accelerate researches for the therapeutic use of phenoxodiol as chemosensitizer in platinum- and taxane-based chemotherapy to cure recurrent ovarian cancer [67].

More than a decade ago, *in vivo* animal models of carcinogenesis revealed the chemopreventive effects of genistein (or isoflavone rich diet) [69,70]. In recent *in vivo* studies, the efficacy of genistein/isoflavone as sensitizing agent in association with chemotherapeutic drugs was also reported [71,72]. Earlier in 1992, soy containing diets was demonstrated to reduce the severity of prostatitis in rats and prevented prostate adenocarcinoma in rat carcinogenesis models [69,70]. In PTEN (phosphatase and tensin homolog) heterozygous (+/-) mutant mice (mPTEN), a model for endometrial carcinoma, it was shown that genistein inhibited PTEN-related tumorigenesis [73]. In a different study, it was reported that soy-derived isoflavones (0.1 mg/day given intra-peritoneally) significantly inhibited growth of T-ALL (T-cell Acute Lymphoblastic Leukemia)-derived cell lines and their infiltration into various organs in NOD/SCID (severe combined immunodeficient) mice [74]. However, in a rat model of colon cancer, adverse effects of genistein have been reported [28]. The results of this study “emphasize that the biological effects of genistein may be organ specific, inhibiting cancer development in some sites yet showing no effect or an enhancing effect on the tumorigenesis at other sites, such as the colon” [28]. Despite this negative observation, strong experimental data support the protective effects of genistein mainly in prostate and breast cancers.

In combination therapy, genistein significantly enhances the antitumor, anti-invasive, and anti-metastatic activities of chemotherapeutic drug docetaxel, both in cell lines and in a murine model of experimental prostate cancer bone metastasis [38]. The authors demonstrated that enhancement of docetaxel efficacy by genistein involves OPG/RANK/RANKL/MMP-9 pathway and results in the inhibition of osteoclastic activity and prostate cancer bone metastasis. Using the PC-3 orthotropic metastatic mouse model, it was shown that soy isoflavones and irradiation leads to enhanced control of primary tumor growth and metastasis in prostate tumors [75]. These results suggest that therapy against prostate cancer might benefit from genistein treatment when associated to docetaxel or radiotherapy.

From data reported above, convincing evidence emerge on the promising therapeutic applications of genistein in cancer therapy. However, the double-faced behavior of the molecule, inhibition of cell proliferation at high concentrations and activation of estrogen-dependent effects at low concentrations, requires caution in determining therapeutic doses in mono-treatment or in combination with chemotherapy, radiotherapy, and immunotherapy. It is reasonable that, at low concentration, genistein may enhance cytotoxicity of conventional anticancer therapy limiting the harmful side effects often observed in patients treated with massive doses of radiotherapy or cytotoxic drugs. This is the main goal in modern cancer therapy currently under investigation in different clinical trials. Actually, 16 clinical trials for genistein and 28 for isoflavone are running on patients affected by a variety of cancers, including breast, prostate cancers, melanoma, kidney, pancreatic cancers and leukemia (a selection of them is reported in Table 2). Previous phase I clinical trials assessed safety, pharmacokinetic parameters and efficacy of orally administrated isoflavones [76,77,78]. These studies demonstrated that soy isoflavone, or purified soy isoflavone mixture (genistein, daidzein and glycitin), were not toxic, nor genotoxic. In subjects with prostate cancer, plasma concentration of genistein after oral administration (150 mg) ranged from 4.3 to 16.3 μM, a dose compatible with its biological activity *in vitro* [76]. However, in a significant percentage of PBMC isolated from patients (77%–78%), the authors observed an increase in the pattern of tyrosine phosphorylation after 6 hr of genistein ingestion, when its plasma concentration was elevated. The predominant band showed a molecular weight of about 60 kDa [76]. This study reflects the potential opposing effects associated to the same molecule depending on the *in vitro* versus *in vivo* experimental model.

To confirm the *in vivo* chemopreventive efficacy of genistein, the molecule was given as dietary supplement in a clinical trial in women at high risk for breast cancer. Other clinical trials are recruiting subjects to study genistein as dietary supplementation in treating patients undergoing radiotherapy for bone metastasis and in patients who are undergoing surgery for bladder cancer. Aim of these studies is to prevent cancer recurrence after surgery or radiotherapy using genistein as dietary supplement. Active but not still concluded clinical trials are using dietary supplement of genistein as promising sensitizer agent associated with interleukin-2 in patients with metastatic melanoma or kidney cancer and in association with gemcitabine and erlotinib (TK inhibitor) in patients with locally advanced or metastatic pancreatic cancer (Table 2). Isoflavone genistein, given as dietary supplement, is under investigation in patients with stage II, III or IV prostate cancer and combined with gemcitabine in treating women with stage IV breast cancer. These studies are completed even if there are no published results. However a phase II clinical study, to investigate modulation of serum PSA levels in patients with prostate cancer after soy isoflavone supplementation (Novasoy), showed a decline of serum PSA levels without any toxicity [79]. Finally, an interesting approach has been adopted in an active clinical trial using a B43-genistein immunoconjugate. Similar strategies may optimize genistein specific targets in leukemia cells, without toxicity in normal, non transformed cells. This trial is recruited patients with recurrent ALL and Non-Hodgkin’s Lymphoma (NHL). When conjugated to B43 monoclonal antibody, genistein is able to target B-lineage leukemia that unlike normal cells, over-expresses the target antigen CD19 [80]. Genistein is also conjugated to recombinant EGF to target cancers over-expressing the EGF receptor as in breast cancer [81].

## 5. (-)-Epigallocatechin-3-gallate (EGCG)

Tea, produced from leaves of *Camelia sinensis* is one of the most popular beverages worldwide and a natural dietary source of polyphenols, mainly catechins. (-)-Epigallocatechin-3-gallate (EGCG) is the most bioactive green tea catechin, as demonstrated in several cell culture studies [82], animal models and epidemiological evidences [83] (Table 1).

To date, the large number of studies on the anticancer properties of EGCG regards its chemopreventive effects. Similarly to other phytochemicals, EGCG modulates different signaling pathways responsible for its chemopreventive and potential chemotherapeutic activity against cancer cells. EGCG, like other flavonoids, is a prototype of phytochemical with multiple effects on intra-cellular signaling. Its antioxidant effects in protecting against oxidative damage of DNA, lipids and proteins, potentially associated with cancer development, has been largely investigated [82]. However, this issue is partially controversial. In fact, EGCG significantly reduces plasma levels of oxidative biomarkers in animal models, but limited efficacy has been proved in humans [84,85]. Moreover, ROS production in cell culture medium by EGCG promoted DNA damage in rodent macrophage-like RAW 264.7 and human promyelocytic leukemic HL-60 cell lines. These pro-oxidant effects of the molecule are dose dependent since human lymphocytes treated with low concentration (from 10^−8^ to 10^−5 ^M) are protected against DNA strand breakage, while the opposite occurs with high doses of EGCG (10^-3^M) [86]. This study is a further example of controversial results when *in vitro* and *in vivo* studies involving natural phytochemicals are compared. In this respect, a recent review suggested a causal association between green tea and liver damage [38]. The hepatotoxicity must probably be attributed to EGCG or its metabolites which, under particular conditions related to the patient's metabolism, can induce oxidative stress in the liver [38]. Serum concentrations of EGCG achieved after tea drinking range between 0.1–1 μM [87], a dose associated with a pro-apoptotic effects in human B-CLL (B-cells chronic lymphocytic leukemia) [88], even if tens micromolar concentration of EGCG are usually necessary in cell culture studies to observe pro-apoptotic effects.

To explain the multiple biochemical targets for tea polyphenols in cell culture studies, it has been speculated that catechins have functional and structural similarity with chaperones: in silico studies revealed several conformations of EGCG, (-)-epicatechin gallate and (-)-epigallocatechin able to interact with cellular components (double strand DNA, RNA, lipids and proteins) [89]. In particular, direct EGCG interactions with nucleic acids are confirmed by surface plasmon resonance assay and cold spray ionization-mass spectrometry [90].

Induction of apoptosis and cell cycle arrest by EGCG is shown in different transformed cell lines, derived from solid tumors (lung, colon, pancreas, and skin) and human leukemia, without affecting normal cells. These biological activities are explained by direct or indirect inhibition of different tyrosine and serine-threonine kinases involved in cell cycle regulation or apoptosis (cyclin-dependent kinases, membrane receptors EGF-R/HER-2, VEGF-R1/R2, IGF/IGF-1, cytoplasmic kinases PI_3_K/AKT and MAPK) [82]. Recently, 67 kDa laminin receptor (67LR) has been identified as a cell surface receptor for EGCG that mediates its anticancer activity. Indeed, expression of 67LR confers EGCG responsiveness to tumor cells, without affecting normal cells, like fibroblasts or PBMC [15]. These authors demonstrated that EGCG induced the disruption of stress fibers and decreased the phosphorylation of the myosin II regulatory light chain (MRLC) at Thr18/Ser19, which is necessary for cell division. Cancer cells were transfected with short hairpin RNA (shRNA) expression vector to downregulate 67LR expression. When the 67LR was silenced, the suppressive effect of EGCG on the MRLC phosphorylation was significantly attenuated. These results suggest that EGCG inhibits cell growth by reducing the MRLC phosphorylation and this effect was mediated by 67LR [91,92].

Using a combination of nuclear magnetic resonance, binding assays,fluorescence polarization assay, and computational docking studies, Leone *et al.* found that EGCG and black tea theaflavinsare very potent inhibitors (*K*_i_ in the nanomolar range) of theanti-apoptotic factors, Bcl-X_L_ and Bcl-2, suggesting a direct binding of EGCG to the in BH3 pocket of these proteins, at concentrations very close to physiological plasma levels, supporting a realistic use of EGCG as anticancer and pro-apoptotic drug in humans [93]. On the other hand, a recent study by Golden *et al.* [94] revealed a side effect of EGCG as an antagonist of bortezomib, a novel proteasome inhibitor in clinical use for MM. This result indicates that green tea polyphenols can be able to block the therapeutic efficacy of some anticancer agents and suggest that consumption of green tea products may be contraindicated during cancer therapy with bortezomib.

In commenting the clinical and preclinical studies based on EGCG administration, it is necessary to underline that a clear cause-effect relationship between amounts of green tea supplemented and protective effects has not been established. Most studies tested green tea in the form of a brewed beverage, rather than in capsule form. One cup of tea contains approximately 50 mg of caffeine and 80–100 mg of polyphenol content, depending on the strength of the tea and the size of cup. Many studies examined the effects of habitually drinking anywhere from 1–10 cups per day (or greater). In capsule form, there is considerable variation in the amount of green tea extract (GTE), ranging from 100 to 750 mg per capsule. Currently, there is no established recommended dose for GTE capsules. Polyphenon E, (decaffeinated green tea catechin mixture) prepared with a standardized dose of EGCG, is usually administered in the range of 0.4–2 g twice a day to 3–6 subjects per dose level.

Initial studies utilizing green tea polyphenols extract (GTP) have focused on the potential chemopreventive activity of EGCG in animals and humans. Earlier in 1992, an anti-skin tumor-initiating effect of EGCG was reported in Sencar mice [95]. The application of EGCG before challenge with 7,12-dimethylbenz[*a*]anthracene as tumor initiator, resulted in significant reduction both in percentage of mice with tumors and number of tumors per mouse compared with controls. Direct application of GTP in humans prevented ultraviolet (UV)-B-induced cyclobutane pyrimidine dimers, considered to be mediators of UVB-induced immune suppression and skin cancer induction [96]. In combination protocols with conventional chemotherapeutic drugs, tamoxifen plus EGCG showed a synergistic cytotoxic effect towards ER-negative breast cancer cells [97]. In athymic nude female mice implanted with MDA-MB-231 breast cancer cells, the same treatment reduced tumor volume of 71% compared with vehicle control, whereas tumor weight was decreased by 80% [97]. Given these promising results, a phase I clinical trial has been conducted to prove the effectiveness of GTE in patients with advanced solid tumors who were refractory to standard therapy (Table 2). A different phase II clinical trial, actually ongoing, is testing the effect of Polyphenon E administration on tumor cell of breast cancer patients in the interval between biopsy and surgery. The investigators’ primary objectives are to determinate the molecular effects of Polyphenon E in breast tumor cells such as c-Met oncogene expression/phosphorylation levels and PI_3_K and MAPK activations. Secondary objectives include serum biomarkers and effects on other tissues. Evaluation of the safety and tolerability of Polyphenon E in these subjects represent an additional aim to be pursued (Table 2). A different study is actually recruiting patients with breast cancer. The investigators hypothesize a reduction in proliferation and/or an increase in apoptosis in cancer cells after a short-term treatment with EGCG. This clinical trial is testing the beneficial effects of green tea (capsule) in biopsy tissues. Green Tea Catechins (GTCs) administration to TRAMP mice, a model of spontaneous development of prostate cancer, results in a substantial delay of tumor progression in 80% of the animals [98]. Based on this evidence, oral administration of GTCs (600 mg/die) in men with high grade prostate intraepithelial neoplasia resulted in only 3% development of prostate cancer after one year of follow up in men taking EGCG capsule, compared to 30% treated with placebo [99]. The same authors identified by qPCR an 8-genes signature that significantly discriminated benign tissue from prostate cancer in both humans and TRAMP mice model [99]. Chow *et al.* [100] performed a phase I pharmacokinetic study to determine the systemic availability of green tea catechins after single oral dose administration of EGCG and Polyphenon E in 20 healthy subjects (five subjects/dose level), randomly assigned to one of the dose levels (200, 400, 600, and 800 mg based on EGCG content). They reported no significant differences in the pharmacokinetic characteristics of EGCG at 800 mg dose versus Polyphenon E administration. The authors concluded that the two catechin formulations resulted in similar plasma EGCG levels. Based on these pre-clinical studies, Pisters *et al*. [101] designed a phase I Trial to determine the maximum-tolerateddose, toxicity, and pharmacology of GTEin 49 patients with solid tumors. Dose-limiting toxicitieswere caffeine related and included neurologic and gastrointestinaleffects. The maximum-tolerated dose of GTE was 4.2 g/m^2^ once daily, or 1.0 g/m^2^ three times day. No major responses occurred. Ten patients with stable disease completed six months of GTE. They concluded that a dose of 1.0 g/m^2^ (equivalent to 7–8 Japanese cups consisting of 120 ml of green tea three times daily) can be recommendedfor further studies. A phase II clinical trial was conducted by the North Central Cancer Treatment Group [102] which evaluated 42 patients manifesting progressive PSA elevation with hormone therapy (androgen independent metastatic prostate carcinoma). Patients received 6 gr/day of green tea in 6 divided doses. They were monitored monthly for response and toxicity. A single patient showed tumor response, defined as a decline ≥50% in the baseline PSA value. This response was not sustained beyond two months. At the end of the first month, the median change in the PSA value from baseline for the cohort increased by 43%. Green tea toxicity (nausea, emesis, insomnia, fatigue, diarrhea, abdominal pain, and confusion) occurred in 69% of patients. In a recent study involved 26 men with positive prostate biopsies and scheduled for radical prostatectomy, patients were treated with daily doses of Polyphenon E (800 mg of EGCG for a total of 1.3 g of tea polyphenols) before radical prostatectomy occurred. Serum biomarkers (hepatocyte growth factor, HGF; vascular endothelial growth factor, VEGF; insulin-like growth factor, IGF-I; IGF binding protein-3, IGFBP-3; and PSA) were analyzed before initiation of the study and on the day of prostatectomy. The results support a potential role for Polyphenon E in the treatment (or prevention) of prostate cancer because the authors found significant reduction in serum levels of PSA, HGF, and VEGF [103]. Shanafelt *et al.* conducted a study on clinical effects of oral GTE administration in six patients with low grade B-Cell malignancies [104], reporting a positive response in four subjects shortly after “self-initiating” GTE therapy. Therefore, the same group started a phase I/II study enrolling 33 previously untreated patients with asymptomatic Rai stage 0 to II CLL to test the efficacy of Polyphenon E [105]. Patients who received Polyphenon E with a standardized dose of EGCG (range 400 to 2,000 mg/day) evidenced minor adverse effects with one patient obtaining partial remission. In addition, 11 patients (33%) showed a 20% reduction in absolute lymphocyte count, and 11 out of 12 (92%) patients with palpable adenopathy experienced at least a 50% reduction of all nodal areas during treatment. After one month of treatment, plasma EGCG levels ranged from 2.9 to 3.9 ng/mL. This encouraging study anticipated a phase II clinical trial to evaluate efficacy of EGCG at 2,000 mg dose, twice a day (Table 2). A phase II clinical trials with Polyphenon E (800 to 1,200 mg EGCG daily for 14–28 days) was also sponsored by University of Wisconsin in patients with non metastatic bladder cancer to compare levels of EGCG in non malignant bladder tissue versus malignant bladder tissue in these patients (Table 2). Secondary objective was the dose–response modulation of surrogate-endpoint biomarkers (e.g., PCNA, MMP2. clusterin, VEGF, p27) in malignant and non malignant samples of bladder tissues and changes in serum levels of IGF-1 and IGFBP-3 after administration of Polyphenon E. No results have been published yet. Finally, a phase II randomized trial is evaluating the effects of six cups of green tea and decaffeinated black tea versus water in 180 patients with prostate adenocarcinoma scheduled to undergo prostatectomy (Table 2). This study aimed to compare levels of prostate cancer biomarkers (PSA) versus tumor development and progression assessed by biopsy. Genotype and gene expression of metabolizing enzymes (COMT, UGT and SULT) have been also evaluated in patients biopsies. Results are not available yet. Given the potential of Polyphenon E to improve chemotherapeutic drugs effects, a phase I/II clinical Trial was initiated in patients with advanced non small cell cancer (NSCLC) treated with novel drug Erlotinib (EGFR/TyrK inhibitor). Combination of Erlotinib with Polyphenon E will be evaluated also in preventing cancer recurrence in former smokers who have undergone surgery for bladder cancer. This trial is enrolling patients (Table 2).

## 6. Lycopene

Lycopene (ψ,ψ-carotene) is the most abundant carotenoid in tomato *Lycopersicon esculentum* L. From a chemical point of view, it is a tetraterpene hydrocarbon extremely lipophlic (Table 1) synthesized by plants and some microorganisms [106]. In nature, lycopene is essentially present as all-trans-isomer. However, the instability of double bounds to light, heat or chemicals induces isomerization [107,108] with the production of a mixture of *cis*-isomers which are commonly found in human serum and tissues [106,109]. Differently than other plant carotenoids possessing vitamin A-like function for the presence of the b-ionone ring, lycopene, lacking this structure, does not have known physiological function in humans, except for its strong antioxidant activity. In fact, the molecule is able to efficiently quench singlet oxygen (^1^O_2_) because the presence of several conjugated double bonds [110], and to scavenge free radicals. It has been demonstrated that a single lycopene molecule can scavenge more than one ROS, due to the presence of the long polyene chain [111]. The antioxidant properties of lycopene have been largely proved in cell culture at micromolar concentrations on several targets, including nitration of protein, DNA damage by hydrogen peroxide and peroxynitrite. In addition, lycopene has been reported to up-regulate the ARE (antioxidant response element)-dependent transcription of antioxidant enzymes, such as superoxide dismutase-1, catalase, epoxide hydrolase and transferrin under the control of Nrf2 transcription factor (reviewed in [106]). However, a recent review focused on human and animal trials testing lycopene, or lycopene-containing extracts, suggests that limited evidence exists for the "lycopene antioxidant hypothesis" as an *in vivo* mechanism of action. Some of the biological activity of the molecule can be attributed to its metabolic products (lycopenoids) [112].

Lycopene concentration in raw tomatoes ranges between 8.8–42 μg/g wet weight. These values increase to 86–100 μg/g and 63–131 μg/g for tomato juice and tomato sauce, respectively [28]. Therefore, the process of cooking usually makes lycopene more bioavailable due to its release from the matrix into the lipid phase of the meal [113]. *Cis-*isomers of lycopene are probably better absorbed than the *all-trans* form. This is due to the shorter length of the *cis-*isomer, the greater solubility of *cis*-isomers in mixed micelles, and the lower tendency of *cis*-isomers to aggregate [113]. All-trans lycopene accounts for 79% to 91% and *cis* isomers for 9% to 21% of total lycopene in tomatoes, tomato paste, and tomato soup. Lycopene concentrations in the serum of men range between 0.60 and 1.9 nmol/mL, with 27% to 42% all-trans lycopene and 58% to 73% *cis*-isomers. In striking contrast with foods, all-*trans* lycopene accounts for only 12% to 21% and *cis* isomers for 79 to 88% of total lycopene in benign or malignant prostate tissues [114]. From these and other data derived that, in healthy human subjects treated with increasing doses of lycopene ranging from 10 to 120 mg, maximum total lycopene concentration was reached between 15.6 and 32.6 h with serum levels ranging between 0.075–0.210 μM [115].

Lycopene bioavailability represents a key issue to explore the chemopreventive and chemotherapeutic properties of the molecule. The preferential *in vivo* bio-accumulation of lycopene isomers within prostate issue [116] suggested its potential involvement against prostate cancer. Several excellent reviews have been published on the antiproliferative and pro-apoptotic activity of lycopene on cell lines derived from human prostate cancer [106,117]. At non physiological concentration, lycopene triggers apoptosis activating the intrinsic pathway involving mitochondrial function, cytochrome C release and exposure to Annexin V. The antiproliferative activity of lycopene is mostly identified with the ability of the molecule to block the cell cycle at G0/G1 phase. This effect has been observed in prostate cancer, hepatocyte, and breast cancer cell lines [106]. It is worthwhile to note that contradictory results obtained by different groups on the same cell lines can be attributed to concentrations applied which, in some cases, differ of several orders of magnitude. In fact, a recent study which employed various different cancerous and noncancerous cell types, treated with lycopene from 0.0001 to 10 μM for 24, 48, and 72 h, indicated that lycopene, at the physiological range, does not significantly affect cell proliferation, suggesting more careful investigations [118].

Since the publication of epidemiological data supporting the idea that consumption of lycopene rich diet lowers risk of prostate cancer [58,119], many studies supported the potential use of the molecule as chemopreventive agent based on data primarily coming from animal studies. However, also in this case, as for cell lines discussed above, a unanimous consensus has not been reached. As an example, in a xenograft model of BALB/c nude mice implanted with DU145 prostate cancer cells, administration of 100 and 300 mg/Kg of lycopene reduced tumor growth of 55.6% and 75.8%, respectively [120], while, in a different model represented by PC-346 C orthotopic mouse no effect was observed at a lower dose (5 or 50 mg/kg) [121]. Association with α-tocopherol also slightly increased necrotic area in MatLyLu Dunning prostate tumor model [122]. A more recent study reports that lycopene inhibits experimental metastasis of human hepatoma SK-Hep-1 cells in athymic nude mice [123]. However, also this study has been partially questioned [124]. Despite these controversial data in animal model, the consistent number of epidemiological studies showing an inverse relationship between tomato/lycopene intake and risk of incurring in several types of cancer [125], stimulated an investigation from FDA for qualified health claims regarding tomatoes, lycopene and cancer risk. The FDA found no credible evidence to support an association between lycopene intake and a reduced risk of prostate, lung, colorectal, gastric, breast, ovarian, endometrial, or pancreatic cancer. The FDA also found no credible evidence for an association between tomato consumption and a reduced risk of lung, colorectal, breast, cervical, or endometrial cancer. The FDA found very limited evidence to support an association between tomato consumption and reduced risks of prostate, ovarian, gastric, and pancreatic cancers [126].

Actually, several phase I and II clinical trials essentially supported by research centers and University are ongoing worldwide (Table 2). Target patients are those with early stage prostate cancer, benign prostatic hyperplasia, before radical prostatectomy for prostate cancer, recurrent or metastatic prostate cancer. In several case (Table 2), combination supplements are tested to ameliorate patient conditions. Usually, these products are currently available on the market, as herb and vitamin supplements, not regulated by the FDA. Each single ingredient has been studied in prostate cancer cells and/or in patients with prostate cancer and at doses included in this supplement, no serious side effects have been reported. In the next future, results of these studies will hopefully clarify the potential pharmacological use of lycopene in cancer therapy.

## 7. Resveratrol

A number of excellent reviews have been recently published on resveratrol (3,4′,5-trihydroxy-*trans*-stilbene), a phytoalexin firstly described as a component in the root of the weed *Polygonum cuspidatum*, whose extracts are very well known in Asian medicine [127,128,129] and also present in grape skin, wine and peanuts. Resveratrol raised his importance as a chemopreventive agent after a paper published in 1997 [130]. However, what seems paradoxical is the discovery that resveratrol was initially present in the scientific literature as cardiovascular protective agent, able to explain the “French paradox”. In fact, the molecule inhibits platelet aggregation, prevents LDL oxidation by means of its antioxidant properties, exerts vasorelaxing effect on animal model (reviewed in [131]). Finally, and more recently, resveratrol showed potential anti-ageing properties [132].

*In vitro*, resveratrol and its analogs trigger numerous intracellular pathways leading to cell growth arrest. As exhaustively reviewed in Table 1 of [128], several molecular targets have been identified in cell lines to support the anticancer activity of resveratrol, including deactivation of AhR (aryl hydrocarbon receptor) and CYP enzymes, activation of phase II detoxification and antioxidant enzymes, inhibition of pro-inflammatory mediators, regulation of cell cycle machinery, activation of pro-apoptotic factors and deactivation of anti-apoptotic compounds, inhibition of metastatic progression [1]. In several, but not all, cellular models, resveratrol causes block of cell cycle progression acting on modulating gene expression. In fact, the molecule is able to down-regulate cyclin D1, D2 and E expression and up-regulating the expression of the Cdk inhibitor p21^WAF1/CIP1^[133]. In parallel, the pleiotropic activity of resveratrol in inducing apoptosis is exerted by triggering both the extrinsic, death receptor-mediated, pathways and the intrinsic, mitochondrial pathways. In the first case, resveratrol induces apoptosis in colon cancer cell lines by redistribution of death receptors, CD95 (Fas/Apo-1) and TRAIL (TNF-Related Apoptosis-Inducing Ligand), into lipid rafts. This effect sensitizes these tumor cells to death receptor-mediated apoptosis. Therefore, resveratrol does not enhance the number of death receptors at the surface of tumor cells but induces their redistribution into lipid rafts and facilitates the caspase cascade activation in response to death receptor stimulation [134]. Alternatively, resveratrol has recently been reported to suppress expression of anti-apoptotic proteins, such as survivin, Bcl-X_L_ and Mcl-1 in NHL and MM cell lines [135]. Inhibition of Bcl-X_L_ expression by resveratrol was mediated by inhibition of the ERK1/2 pathway (reviewed in [135]).

Several *in vivo* studies, recently reviewed [127,129,136], sustain resveratrol efficacy in inhibiting or retarding tumor growth and/or progression in animal models inoculated with malignant cell lines, or treated with tumorigenesis-induced drugs (benzo-[*a*]pyrene,7,12-dimethylbenz[*a*]anthracene, azoxymethane). However, lack of *in vivo* efficacy of resveratrol was reported by others [137]. The tumor preventive activity of resveratrol has been confirmed in a wide variety of experimental tumors including skin, liver, colon, breast, lung, and esophageal cancers. In general, the majority of these studies agree on the ability of the molecule to prevent chemically-induced malignancies and of reducing the efficiency of experimental protocols of tumorogenesis (reviewed in [136]).

Sinclair’s group and others described the anti-ageing properties of resveratrol [138]. The molecule can extend the life span of yeast, worms, flies, and fish and mitigates the metabolic dysfunction of mice fed high-fat diets. Resveratrol appears to mediate these effects partly by activating sirtuin (SIRT1), a deacetylase enzyme that regulates the activity of several transcriptional factors and enzymes responsive to nutrient availability [132]. The pro-survival properties of resveratrol have been confirmed by a recent paper showing its ability to increase aerobic capacity in mice by inducing genes for oxidative phosphorylation and mitochondrial biogenesis in a SIRT1-dependent manner [139]. This appears as a potential contradiction respect to the chemopreventive activity of resveratrol: as an antioxidant, resveratrol counteracts ROS production and inhibit cell growth; however, as an anti-ageing compound, the same molecule increases mitochondrial metabolism and, in turns, ROS production, enhancing cell survival. In addition, while histone deacetylases “inhibitors” represent novel and potent chemotherapeutic drugs [140], resveratrol, apparently, functions in the same way “activating” the same enzymes. As discussed elsewhere [1], the *in vivo* bioavailability of the molecule may explain the controversial data on resveratrol mechanism of action. In fact, dietary resveratrol (up to 25 mg) is rapidly adsorbed and predominantly present in the plasma as glucoronide and sulfate conjugates. In addition, when administered in food, such as wine or grape juice, resveratrol metabolism is significantly inhibited by other polyphenols due to competitive reactions with metabolizing phase II enzymes, resulting in an increased concentration of the free form. Despite this, the free aglycone is almost undetectable in human plasma [1,141]. Therefore, caution is suggested when interpreting the voluminous Literature on anticancer activity of resveratrol, only based on *in vitro* studies on cell lines where the molecule is given at pharmacological concentrations (25–50 μM or higher), as aglycone, a form almost absent in plasma and urine [1]. The explanation for the functional pleiotropy of resveratrol might be found in the values of its circulating concentrations. In fact, the molecule activates *in vivo* SIRT1 at very low concentration (nanomolar range), a value potentially in agreement with resveratrol bioavailability. On the opposite, moving from physiological to pharmacological concentrations, as those employed in the *in vitro* experiments, predominates the anticancer properties of the molecule, such as inhibition of cell growth, activation of apoptotic events, arrest of cell cycle progression, depending on the cell line employed and the biomarkers analysed.

Authors agree on the conclusion that despite the numerous reports describing the pharmacokinetics of resveratrol in animal model systems, there are only few similar studies in humans to date, and the large part of these data regards the administration of pure or dietary resveratrol (in wine and/or other beverages) in healthy subjects to assess its bioavailability in view of a chemopreventive use of the molecule, more than an its therapeutic use in cancer patients [127,136,142]. As an example, data from a case-control study, analyzed the relation between dietary intake of resveratrol and 50% or greater reductions in breast cancer risk in women with resveratrol consumption from grapes, but not from wine. The inverse relationship between resveratrol and breast cancer risk could not be explained by several potential confounding factors, including alcohol intake, nor was it attributable to a nonspecific favorable effect of fruit on breast cancer risk [142,143]. Similarly, a closed phase I clinical trial on healthy subjects is evaluating the effect of grape-derived low-dose resveratrol on biomarkers related to the Wnt pathway, a key signaling pathway activated in >85% of colon cancers. The same study is evaluating the utility of this approach for colon cancer prevention [142]. Ongoing clinical trials on phase I and II using resveratrol as pure compound or formulation with improved bioavailability (e.g., SRT501 from Sirtris Pharmaceuticals) are summarized in  2.  In  the  first  two  trials  patient  with  colon  cancer  and  resectable  colorectal  cancer  respectively  will  receive  oral  doses  of  resveratrol  to  examine  its  effects  directly  on  colon  cancer  and  normal  colonic  mucosa  and  determine  changes  in  several  biomarkers  such  as  Wnt  and  COX-2  expression  Ki67  labeling  index.  The  toxicity  profile  of  the  molecule  will  also  be  evaluated.  In  the  two  studies  sponsored  by Glaxo Smithkline (Table 2), the safety, pharmacokinetics and pharmacodynamics of SRT501 will be assessed in subjects diagnosed with colorectal cancer and hepatic metastases or MM. In the latter case, the efficacy of SRT501 will be evaluated in monotherapy or in combination with bortezomib.

## 8. Conclusions

Here we reviewed the anticancer activity of a group of phytochemicals representing good candidates for chemopreventive and chemotherapeutic applications. They have been selected among many others available in the literature to play the “truth or dare” game: do they really possess efficacy in cancer therapy? All compounds reviewed in the present work have in common the presence in the diet and a well documented anticancer activity in experimental models, namely cell cultures and animal models of induced carcinogenicity. These molecules are largely cited in the current literature and their activity is often associated with the term chemoprevention. However, we clearly showed here that the translation from bench to clinics can be applied only to a limited number of them (Table 2). We underlined some critical issues generating controversy, such as the different order of magnitude of doses employed and the wide spectrum of biological processes affected by these compounds, ranging from cancer to ageing. To give a rationale explanation on how the same molecule can trigger pathways linked to different physio-pathological conditions, as in the case of resveratrol, is not an easy exercise. To increase confusion, in clinical trials referring to phytochemicals, it is not always clear when they are tested as potential chemopreventive or chemotherapeutic agents. The difference existing between primary and secondary chemoprevention and chemotherapy has been authoritatively defined [2,144,145]. Chemoprevention should be referred to “healthy” people who agree to receive a therapeutic treatment lasting, perhaps, years; therefore, safety represents a key issue in chemoprevention, since lack of toxicity or undesirable side effects are mandatory. In addition, practical considerations suggest only oral administration of the active principle as pills, capsules or similar formulations. On the opposite chemotherapy is applied to patient who already developed tumors and/or invasive cancers. In this case, drugs are given at pharmacological doses and their intrinsic toxicity is evaluated by a strict benefit-versus-risk analysis. Studies summarized in Table 2 essentially aim to evaluate how selected phytochemicals should be given (e.g., by mouth or injected into the blood), how often and what dosage is safe. When administered *per os*, phytochemicals can be found in free form in the blood only if they are taken at pharmacological doses (hundreds of milligrams). In fact, only in this case, they saturate the metabolic pathways of conjugation (methylation, sulfatation and glucuronidation) and are potentially bioavailable and biologically active [146]. Alternatively, they must be administered by intravenous injection to avoid that the formation of conjugates can reduce the bioavailability of the active moiety and dramatically alter their pharmacological properties. The correct dose will also determine the primary site of metabolism, with high doses primarily metabolized in the liver and low doses in the intestine. We listed above the attributes which a putative phytochemical compound should respect to be applied in chemoprevention. From this, it appears not easy to determine the adequate dose which is not neutralized by metabolizing enzymes, and, in parallel, is not too high to cause toxicity in humans. These obstacles are bypassed when the active compound is employed as chemotherapeutic agent against specific types of cancers. In this case, higher doses and a greater toxicity are acceptable, since the target is represented by patients with advanced cancers. Randomized phase III clinical trials employing phytochemicals as tested drugs are devoted to provide these data enrolling a large number of cancer patients. However, until now, only in few cases, e.g., curcumin (Table 2), trials with phytochemicals reached phase III.

Finally, in the current attempts to translate the anticancer efficacy of phytochemicals into clinics, we cited several examples of combination therapy (Table 2), where anticancer compounds are given in association with well-known drugs currently used in chemotherapy. From a pharmacologic point of view, this strategy presents several advantages. Phytochemicals are functionally pleiotropic: we clearly showed that they possess multiple intracellular targets, affecting different cell signaling processes usually altered in cancer cells, with limited toxicity on normal cells. Targeting simultaneously multiple pathways may help to kill cancer cells and slow drug resistance onset. In addition, if proven, the association of pure or synthetic analogs of phytochemicals with chemotherapy or radiotherapy may take advantage of the synergic effects of the combined protocols, resulting in the possibility to lower doses and, consequently, reduce toxicity.

The results of the ongoing clinical trials may provide a foundation for designing future large-scale clinical trials to ascertain the full chemopreventive and chemotherapeutic efficacy of phytochemicals.
